# Low field slice-selective ZTE imaging of ultra-short $T_2$ tissues based on spin-locking

**DOI:** 10.1038/s41598-023-28640-x

**Published:** 2023-01-30

**Authors:** Jose Borreguero, Fernando Galve, José M. Algarín, José M. Benlloch, Joseba Alonso

**Affiliations:** 1 Tesoro Imaging S.L., 46022 Valencia, Spain; 2 Institute for Molecular Imaging and Instrumentation, Spanish National Research Council, 46022 Valencia, Spain; 3 Institute for Molecular Imaging and Instrumentation, Universitat Politècnica de València, 46022 Valencia, Spain

**Keywords:** Biomedical engineering, Magnetic resonance imaging

## Abstract

Magnetic Resonance Imaging of hard biological tissues is very challenging due to small proton abundance and ultra-short $T_2$ decay times, especially at low magnetic fields, where sample magnetization is weak. While several pulse sequences, such as Ultra-short Echo Time (UTE), Zero Echo Time (ZTE) and SWeep Imaging with Fourier Transformation (SWIFT), have been developed to cope with ultra-short lived MR signals, only the latter two hold promise of imaging tissues with sub-millisecond $T_2$ times at low fields. All these sequences are intrinsically volumetric, thus 3D, because standard slice selection using a long soft radio-frequency pulse is incompatible with ultra-short lived signals. The exception is UTE, where double half pulses can perform slice selection, although at the cost of doubling the acquisition time. Here we demonstrate that spin-locking is a versatile and robust method for slice selection for ultra-short lived signals, and present three ways of combining this pulse sequence with ZTE imaging of the selected slice. With these tools, we demonstrate slice-selected 2D ex vivo imaging of the hardest tissues in the body at low field (260 mT) within clinically acceptable times.

## Introduction

Zero Echo Time pulse sequences, otherwise known as Zero TE or ZTE, are designed to capture the weak and short-lived signal emitted by hard biological tissues and solid-state matter in Magnetic Resonance Imaging (MRI) scanners^1,2^. ZTE sequences are particularly suitable for ultra-short (sub-milli-second) $T_2$ or $T_2^*$ MRI^3^, and have been successfully used for a wide variety of applications^4^, including imaging of tendons and bones^5,6^, teeth^7–9^, myelin^10^ or lungs^11^. Two other families of sequences used for MR imaging of short $T_2$ samples are SWeep Imaging with Fourier Transformation (SWIFT^12^) and Ultra-short Echo Time (UTE^13^), although the latter is not suitable when the sample $T_2$ is comparable to or shorter than the time it takes to switch on the encoding magnetic gradient fields. Despite their success at imaging the hardest biological tissues, one limitation shared by both ZTE and SWIFT is that, in their basic forms, they are 3-dimensional and cannot produce a 2D image of a pre-selected sample slice.

The capability to selectively excite a given slice in the sample and obtain a 2D image is an essential part of the general MRI toolbox, even if it was so far out of reach for ultra-short $T_2$ samples. With slice selection, the overall acquisition time for a single slice can be shortened, and 3D aliasing and ringing artifacts from slice to slice are avoided^14^. Besides, slice selection can be critical for quantitative MRI, where slice profile assessment is a concern^15^.

Slice selection typically employs a soft radio-frequency (RF) pulse in the presence of a magnetic gradient perpendicular to the desired plane^16^. This long excitation stage is incompatible with the short-lived signals from hard tissues. Consequently, sequences for MR imaging of ultra-short $T_2$ tissues are inherently volumetric. Slice selection can be realized with UTE by dividing the RF pulse into two halves, which are imaged sequentially and then merged into a unique signal^17^. Unfortunately, UTE is generally unsuitable for samples with $T_2 <1$ ms, slice selection in this way doubles the scan time, and the RF half pulses are sensitive to Eddy currents from the fast switching of the slice selecting gradient, making it challenging even with recent advances^18,19^. For ZTE and SWIFT sequences, we are not aware of any prior work illustrating the possibility of slice selection.

The difficulties are specially severe in the low field regime, where signals are much weaker due to lower equilibrium polarization of tissues. With the idea of developing a replacement for X-ray scanners for wide-spread use in dental clinics in the long term, we have developed a custom-built low-field MRI scanner at 260 mT, where we have previously shown simultaneous 3D imaging of soft and hard tissues with a ZTE sequence^8^.

In this paper, we demonstrate a technique for slice-selective ZTE of ultra-short $T_2$ species, combining a slice-selective block based on spin-locking in the presence of a linear magnetic gradient field^20^, and a ZTE image-encoding sequence. We show that spin-locking is a versatile, robust and well suited complement to ZTE (and potentially other) pulse sequences. During spin-locking, a single-tone RF excitation of amplitude $B_\text {1SL}$ is resonant only with spins in the selected slice, due to the presence of the gradient. This “locks” the selected magnetization to the transverse direction, and slows down the signal loss characteristic time from $T_2$ to $T_{1\rho }$^21^, which can be an order of magnitude longer than $T_2$ for hard tissues (where $T_1\gg T_2$). We call the new sequence PreSLoP (*Preserved Spin-Locked PETRA*), where PETRA stands for Pointwise Encoding Time-reduction with Radial Acquisition and is a well-known ZTE variant where the unavoidable gap at the center of k-space, typical of these acquisitions, is filled in a pointwise fashion^8,22^. PreSLoP includes a preservation pulse^23^ after spin-locking to store the magnetization while the slice and encoding gradients are switched off/on, respectively, allowing for ultra-short $T_2^*$ 2D-MRI, the goal of this work. We also employ a simpler version (DiSLoP, *Direct Spin-Locked PETRA*), where imaging can take place right after slice selection. DiSLoP is suitable only for long and moderately short $T_2$ times. In the following sections, we show control over the position, shape and thickness of the selected slice, and present 2D and 3D ZTE images of ultra-short $T_2$ samples and hard tissues including a cow bone and a horse tooth. We find that PreSLoP features higher immunity to Eddy current effects than DiSLoP and PETRA, and also delivers a higher signal-to-noise ratio (SNR) per unit time.

## Theory

### Spin-locking

Spin-locking (SL) was first reported in the 1950s by Redfield while investigating Nuclear Magnetic Resonance (NMR) saturation in solids^24^. In the late 1970s, Wind et al. used SL as an alternative means of slice selection^20^, which was improved later by Rommel and Kimmich^25^ and is now a mainstay in the field of solid-state NMR^26^. In the context of this work, its main advantage compared to the usual soft pulse approach for slice selection is that in-slice magnetization is kept aligned (locked) with the resonant RF field. Consequently, the transverse magnetization is lost at a rate defined by $T_{1\rho }$, rather than $T_2$. In general, $T_{1\rho }$ is significantly longer than $T_{2}$ (see BPP relaxation theory for liquids^21^), enabling the detection of short-lived tissues and samples whose signal would have faded away after slice selection by common procedures, where spectrally selective RF pulses are typically $>1$ ms. Furthermore, because $T_{1\rho }$ depends on the amplitude of the spin-locking pulse, spin-locking (without gradient) has been often used as a tunable contrast mechanism ($T_{1\rho }$-weighted MRI)^27^.

**Figure 1:**
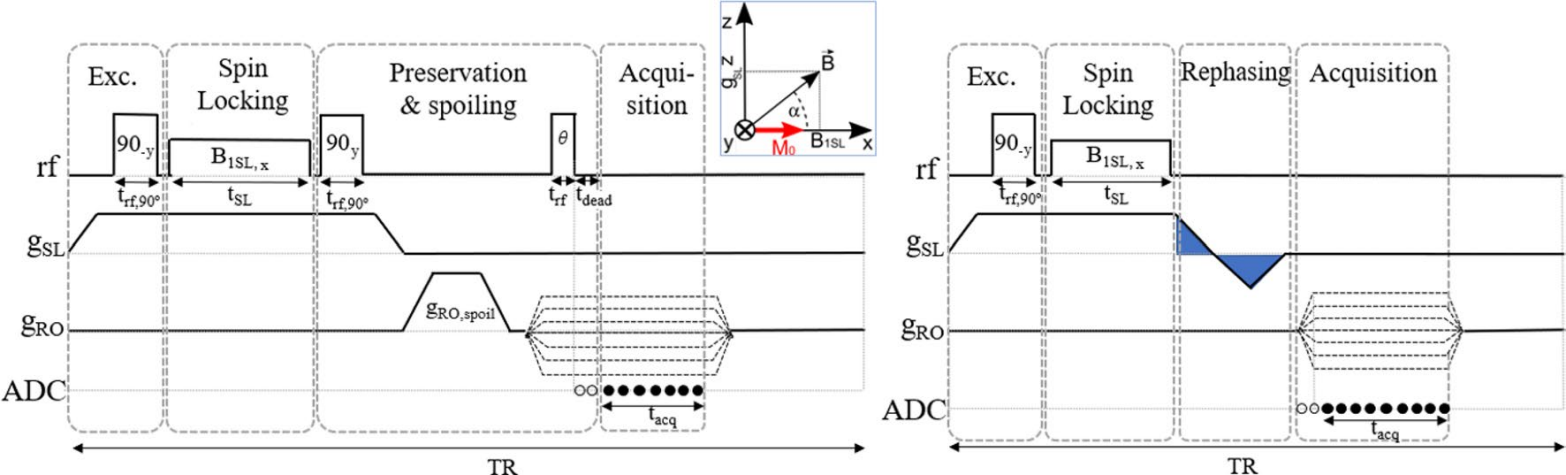
Proposed protocols for 2D imaging of short $T_{2}$ samples: PreSLoP (left) and DiSLoP (right). The empty circles in the ADC (analog-to-digital converter) line indicate the dead time, before the start of the acquisition, which leads to the gap at the k-space center. The inset shows the rotating frame field vectors.

Slice selection through spin-locking works by first applying a gradient $g_\text {SL}$ in the slice direction (*z* in the inset of Fig. 1), and then a hard $90^{\circ }$ pulse which rotates the sample magnetization onto the plane transversal to $B_{0}$. Immediately after this, the RF locking field is pulsed on for a time $t_\text {SL}$. This has strength $B_\text {1SL}$, it is phase-shifted by $90^{\circ }$ with respect to the first excitation pulse so that it is aligned with the precessing magnetization direction ($-y$ in Fig. 1), and it is resonant with $\gamma \left( B_{0} + g_\text {SL} z_{0} \right)$, with $z_{0}$ the position of the slice to be selected. In the inset of Fig. 1 we show the situation for $z_0=0$: spins close to this plane see only a locking field along $-y$, while off-slice spins mostly see a field $g_\text {SL}\cdot z$ along *z*, with a rather abrupt transition between both regimes due to the functional dependence $B(z)=\sqrt{B_1^2+(g_\text {SL} \cdot z)^2}$. Therefore, off-slice magnetization is both homospoiled by the inhomogeneity created by $g_\text {SL}$ and dephased by its intrinsic short $T_{2\rho }$ (the magnetization decay rate in the rotating frame in the plane perpendicular to the SL field), while *in-slice* magnetization is locked by $B_\text {1SL}$ and decays with the much longer $T_{1\rho }$. The magnetization profile after spin-locking has an approximate Lorentzian profile $B_\text {1SL}^2 / [B_\text {1SL}^2+(g_\text {SL}\cdot z)^2]$ with a full width at half maximum (see Supp. Inf. Sect. 1.1)

1 $$\begin{aligned} \Delta z \approx 2B_\text {1SL}/g_\text {SL}. \end{aligned}$$

The SL time required for the slice to be selected, which we arbitrarily define as having achieved such Lorentzian shape up to a $\sim 10\%$ error in an homogeneous sample, can be roughly estimated as

2 $$\begin{aligned} t_\text {SL} \gtrsim \frac{7\pi }{2\Omega _\text {1SL}}\,\text {, where } \Omega _\text {1SL} = \gamma B_\text {1SL} = \gamma g_\text {SL} \Delta z/2, \end{aligned}$$

with $\gamma$ the gyromagnetic factor ($\approx 2\pi \cdot 42$ MHz/T for protons). This is even faster for short $T_{2\rho }$ samples, because of the rapid decay of off-slice contributions. The equations governing the magnetization dynamics are derived in the Supp. Inf. (Sect. 1.1). All in all, the experimental knobs for slice selection are $g_\text {SL}$, $B_\text {1SL}$ and $t_\text {SL}$. In the rotating frame, the longitudinal relaxation time $T_{1\rho }$ is determined by the spectral density of heat-bath fluctuations at the frequency $\gamma \sqrt{B_\text {1SL}^2+(g_\text {SL} \cdot z)^2}$, and the fundamental condition for SL to work is that

3 $$\begin{aligned} T_2^*\ll t_\text {SL}\ll T_{1\rho }, \end{aligned}$$

where $T_2^*$ can be extremely short because it is due to $g_\text {SL}$.

### PreSLoP: Preserved spin-locked PETRA

PreSLoP (Fig. 1, left) is designed for 2D imaging of the hardest biological tissues. This sequence starts with an *excitation block*, where we ramp up the slice selection gradient $g_\text {SL}$ and later transfer the whole sample magnetization to $-y$ with a hard $90^{\circ }_x$ RF pulse (that rotates the magnetization by $90^{\circ }$ around the *x* axis in the frame of reference that revolves at the spin precession frequency). Importantly, this pulse needs to be short and intense enough to span the bandwidth given by $g_\text {SL}$ in the sample volume. Then comes the *spin-locking block*. This is a magnetization preparation block where we switch on $B_\text {1SL}$ along $-y$ for a time $t_\text {SL}$ long enough to dephase off-slice spins. This time can be estimated from Eq. (2) or found empirically if the sample contains ultra-short $T_{2\rho }$ components. For a given slice width, selection is faster for stronger $g_\text {SL}$, which requires also higher $B_\text {1SL}$ strength (Eqs. (1) and (2)). This is important to retain in-slice signal, which decays as $T_{1\rho }$ during SL. Third comes the *preservation & spoiling block*, prior to acquisition. This is designed to prevent $T_{2}^*$ decay with a preservation pulse (hard $90^\circ _{-x}$ RF pulse) directly after spin-locking, placing the in-slice magnetization along *z*. In this way we preserve the coherence while we switch off/on the slice/encoding gradients, which is crucial for 2D imaging of tissues with extremely short $T_2$. During this switching, an additional spoiling gradient pulse can be included to remove remaining off-slice coherences, even if we have found this unnecessary in our system. Finally, in the *acquisition block* we acquire data along radial spokes in k-space following the ZTE procedure, i.e. we ramp up a magnetic gradient $g_\text {RO}$ along the readout direction, and we start the acquisition after its onset. In the acquisition block of PreSLoP, the magnetization can be excited to an arbitrary flip angle $\theta$ and the acquisition starts after the onset of $g_\text {RO}$, as in a standard PETRA sequence. This is an advantage compared to DiSLoP (see below), since the long scan times inherent to PETRA can be partly compensated by using Ernst angle excitations and shortening the repetition time TR^3^. For simplicity, however, we have used $\theta =90^\circ$ throughout the paper.

PreSLoP can be subject to asymmetries and distortions in the slice profile due to $B_0$ drifts caused by Eddy currents, especially for short repetition times (TR). Inverting the polarity of $g_\text {SL}$ between neighboring radial spokes mitigates this effect, because the contributions average out to a large extent (see Fig. S5 in the Supp. Inf. Sect. 2.6). All the PreSLoP images in this paper are therefore taken this way.

### DiSLoP: Direct spin-locked PETRA

DiSLoP (Fig. 1, right) is similar to PreSLoP, but works only with samples whose $T_2$ are not extremely short, longer than units of milliseconds. The preservation & spoiling block is replaced by a *rephasing block*, where we ramp down $g_\text {SL}$ as fast as possible to constrain the $T^{*}_{2}$ decay of in-slice spins. An extra gradient blip - previously calibrated to prevent distortions due to Eddy currents - compensates the in-slice dephasing caused by ramping down $g_\text {SL}$. With DiSLoP, one could start acquiring data while ramping up the gradient, as in UTE, but this would have precluded the performance comparisons we present in Sect. 3.

### Advanced spin-locking schemes

The shape of the slice achieved with SL is determined by an interplay between the spin-locking dynamics and the time envelope of the resonant RF pulse. The diagrams in Fig. 1 show square pulses, which result in roughly Lorentzian spatial profiles (see Supp. Inf. Sects. 1.1 and 1.2). However, it is sometimes convenient to produce sharp rectangular profiles, e.g. for quantitative MRI, so that off-slice contributions do not contaminate the resulting reconstructions^28^. Slice selection based on SL also allows for this, using a single-frequency field with a sinc modulation envelope^29^. Although we have demonstrated the viability of this approach (Supp. Inf. Sect. 2.2), there are two disadvantages with respect to square SL pulses: (i) a sharp square profile requires several sinc lobes, thus lengthening the spin-locking duration, and (ii) the modulation of $B_\text {1SL}(t)$ means $T_{1\rho }$ is given by a sampling of the heat-bath spectral function at locking fields lower than $B_\text {1SL}^\text {max}$, thus leading to a shorter ‘effective’ $T_{1\rho }$, which is the crux of the matter. For this reason, we have used unmodulated locking fields in the remaining studies.

A further interesting possibility is multi-slice selection (see Supp. Inf. Sect. 1.2), which can speed up imaging significantly through the simultaneous excitation and readout of multiple slices^30^. This advantage can be translated to slice selection based on spin-locking by means of a multi-tone excitation resonant with spins at multiple locations.

## Results

We present next our results for structured phantoms and biological samples. Both PreSLoP and DiSLoP contain a slice-selective block followed by an image-encoding PETRA sequence which scans a 2D image of the selected slice. However, we will also use slice selection followed by a 3D image encoding sequence, to show in 3D the image of the selected slice. For this reason we will emphasize in each occasion whether encoding happens in 2D or 3D, by naming e.g. 3D-PreSLoP when we mean that a slice-selective block with preservation is followed by a 3D PETRA image-encoding pulse sequence.

### Structured phantoms

**Figure 2:**
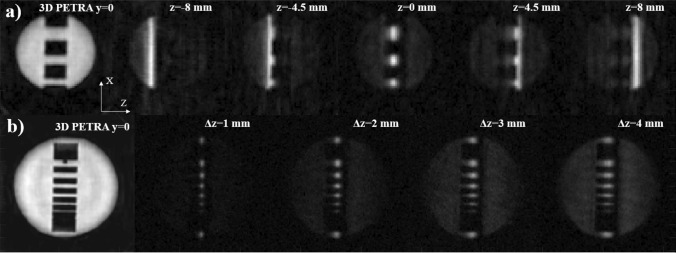
Control over slice selection for two PLA structured phantoms filled with 3 % CuSO$_{4}$ doped water. Leftmost images correspond to PETRA, while the rest are obtained with 3D-DiSLoP, with (**a**) slice positions $z\approx -8$, −4.5, 0, 4.5 and 8 mm, and (**b**) slice thicknesses $\Delta z\approx$1, 2, 3, 4 mm.

Figure 2a shows the $y=0$ slice from a 3D-PETRA acquisition (top left) on a copper sulfate solution in a PLA phantom, along with 3D-DiSLoP reconstructions following the first procedure described in ‘structured phantoms’ in the Methods section. This demonstrates control over the slice position with SL.

Figure 2b shows the $y=0$ slice from a 3D-PETRA acquisition (bottom left) using another PLA phantom, along with 3D-DiSLoP reconstructions following the second procedure described in ‘structured phantoms’ in the Methods section. This demonstrates control over the slice thickness with SL.

**Figure 3:**
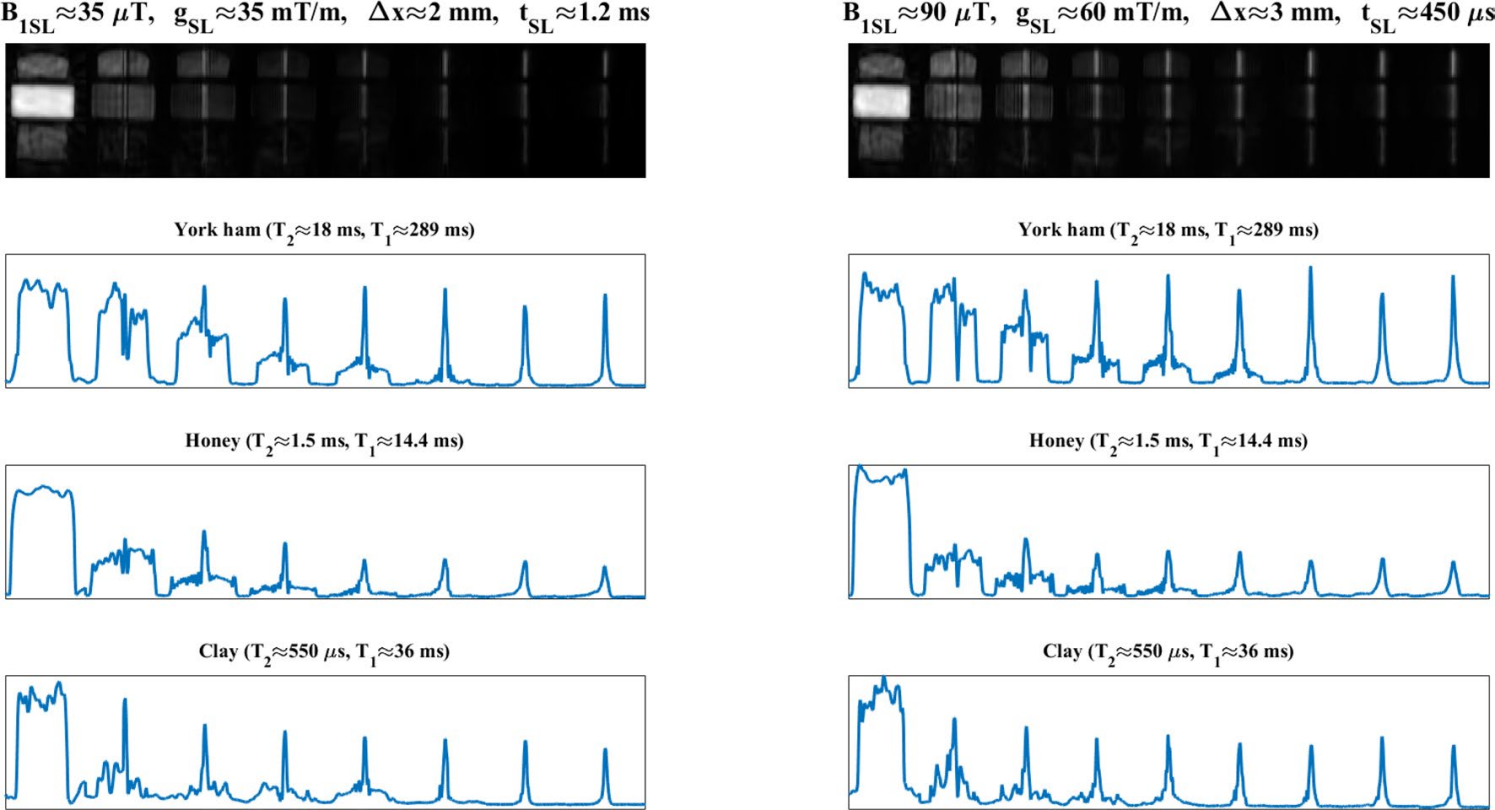
Evolution of the slice selection process as a function of $t_\text {SL}$ for a structured ‘phantom' with York ham, honey and clay. The $T_1$ and $T_2$ times for the samples at 0.26 T are indicated above the corresponding 1D profiles on the three bottom rows, which correspond to the central lines of the samples in the top row images. The slice selection process is depicted for two different sets of SL parameters: $g_\text {SL}\approx 35$ mT/m and $B_\text {1SL}\approx 35\,\upmu \hbox {T}$ (left); and $g_\text {SL}\approx 60$ mT/m and $B_\text {1SL}\approx 90\,\upmu \hbox {T}$ (right).

Figure 3 shows the evolution of the slice-selection process as a function of $t_\text {SL}$ for three different samples, following the last procedure described in ‘structured phantoms’ in the Methods section. For $B_\text {1SL}\approx {90}\,\upmu \hbox {T}$ (right), all three slices (ham, honey and clay) are formed approximately at the same time, and Eq. (2) provides a reasonable estimation regardless of $T_2$ ($t_\text {SL}\approx {450}\,\upmu \hbox {s}$). The situation is qualitatively different for the weaker SL pulse (left, $B_\text {1SL}\approx {35}\,\upmu \hbox {T}$). Here, Eq. (2) predicts $t_\text {SL}\approx {1.2}\,\hbox {ms}$, which is reasonably accurate for ham and honey, but an obvious overestimation for clay, which has a $T_2$ of only ${550}\,\upmu \hbox {s}$. For the latter, the slice formation is accelerated by the rapid $T_2^*$ decay of off-slice contributions (Sect. 1.1). Figure 3 also shows that the time required to select the slice is largely dependent on the amplitude of the SL pulse (note the different time scales on the left and right plots), and the prolonged coherence of in-slice spins (the clay signal lives significantly longer than its ultra-short $T_2$). The notable drop in signal amplitude observed for the honey after the shortest SL pulse is consistent with a proton pool ($\approx 40$ %) that interacts strongly with crystals in the honey and has a $T_2$ around five times shorter than the other $\approx 60$ %^31^.

### Biological samples

**Figure 4:**
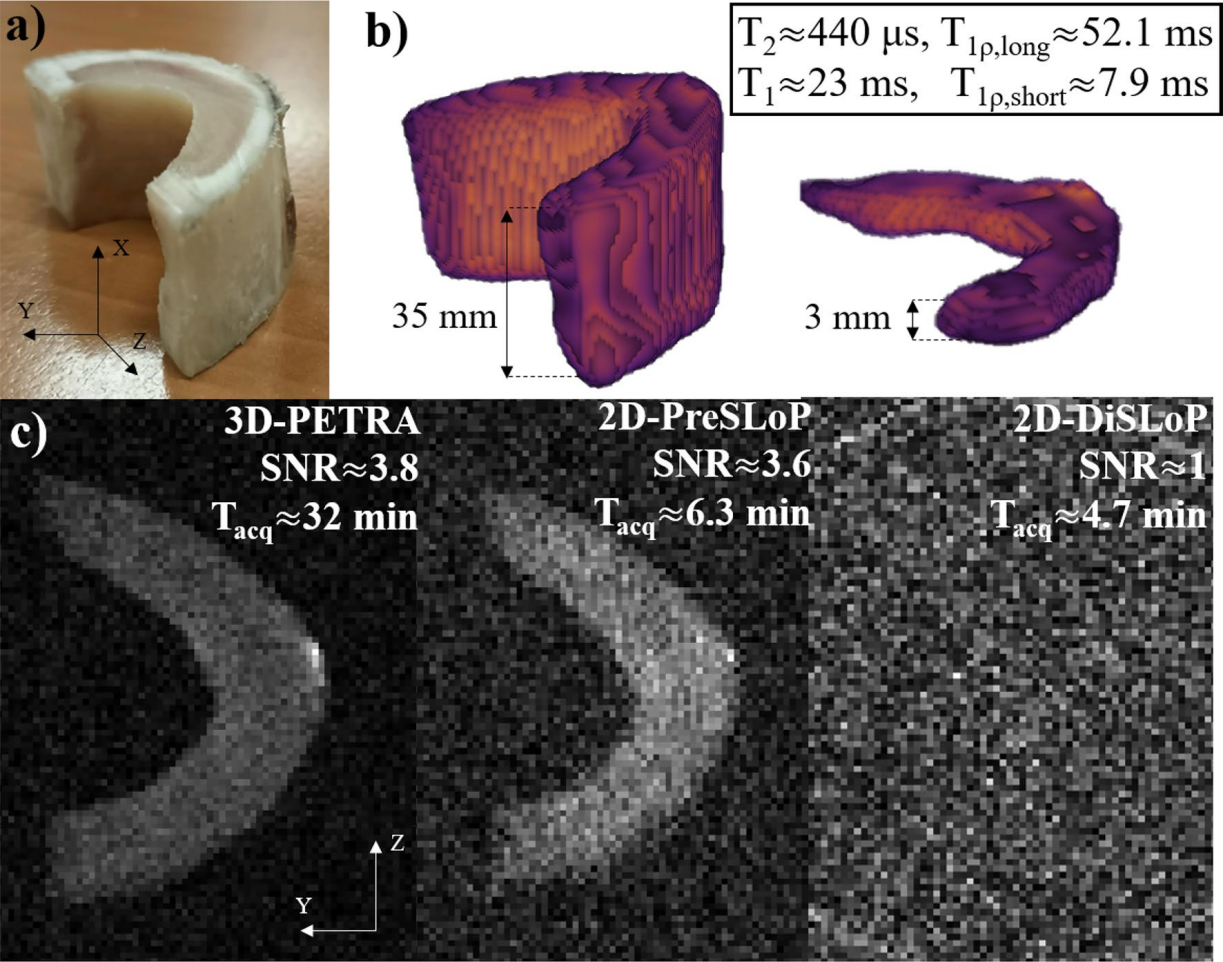
Performance of DiSLoP and PreSLoP for an ultra-short $T_2$ biological tissue (cortical bone). (**a**) Photograph of bovine femur sample. (**b**) 3D-PETRA (left) and 3D-PreSLoP (right). (**c**) 2D images for PETRA (left) with slice resolution $\approx 3$ mm, PreSLoP (middle) and DiSLoP (right) with $\Delta z\approx 3$ mm. We provide $T_{1\rho }$ for both tissues, and the average $T_1$ and $T_2$ values (see Tab. S1).

Figure 4 shows 3D reconstructions of the cow bone using both a standard PETRA sequence, where the whole sample is excited and imaged, and 3D-PreSLoP. Additionally, the bottom row in the figure shows a comparison between 2D images of the central slice ($z=0$, $\Delta z\approx 3$ mm) with 3D-PETRA, 2D-PreSLoP and 2D-DiSLoP. The acquisitions follow the procedures described at the beginning of Sect. 3. Given the ultra-short $T_{2}$ values in the sample, PreSLoP performs significantly better than DiSLoP, where the resulting image is dominated by noise even with our rephasing block of only ${400}\,\upmu \hbox {s}$. Regarding SNR, PreSLoP and PETRA yield very similar results, but the acquisition with the former is $\times 5$ faster. In Sect. 3 we discuss the expected performance of PETRA, PreSLoP and DiSLoP in terms of $t_\text {scan}$ and SNR.

**Figure 5:**
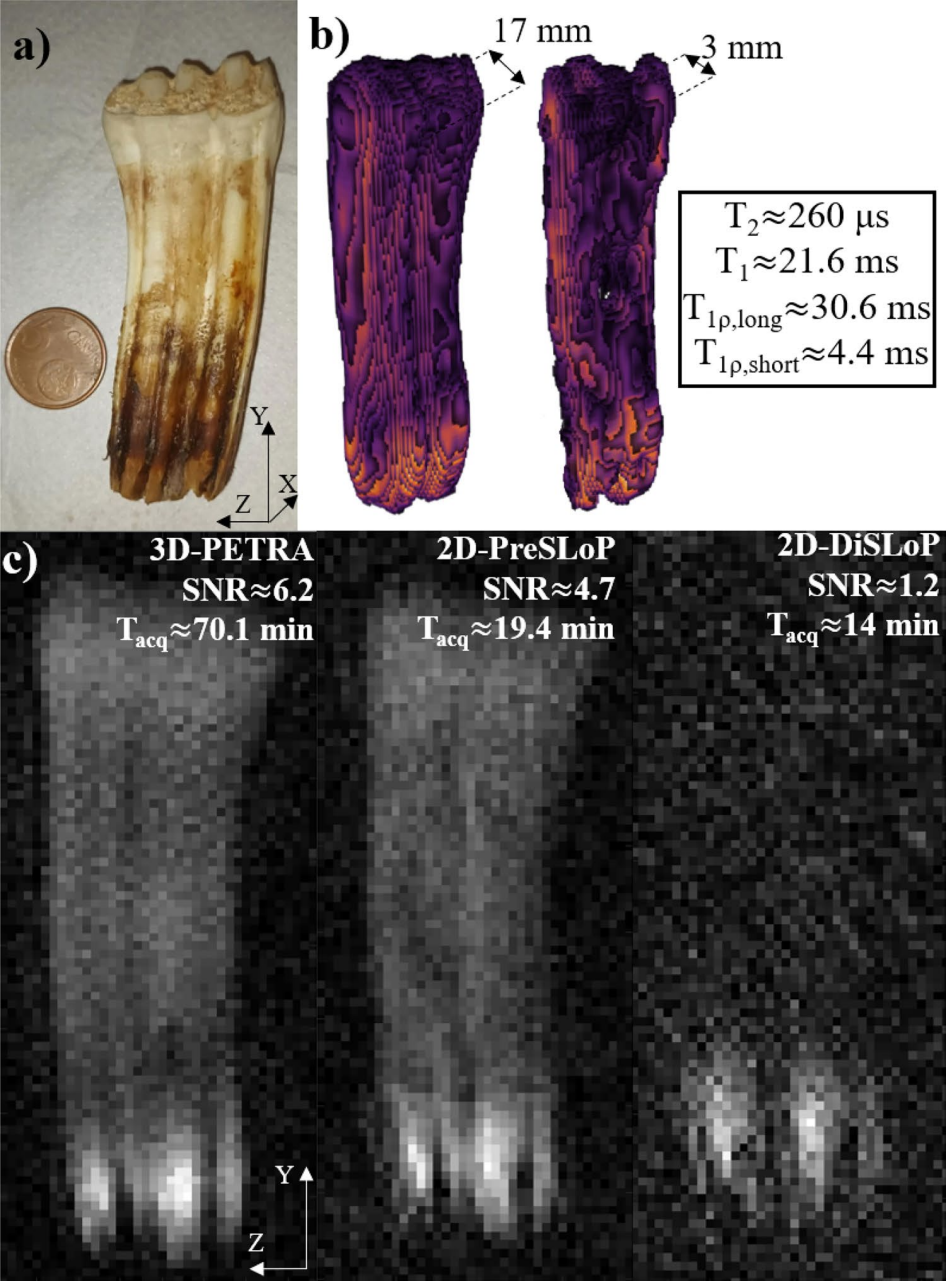
Performance of DiSLoP and PreSLoP for an ultra-short $T_2$ biological tissue (horse tooth). (**a**) Photograph of the sample. (**b**) 3D-PETRA (left) and 3D-PreSLoP (right). (**c**) 2D images for PETRA (left) with slice resolution $\approx 3$ mm, PreSLoP (middle) and DiSLoP (right) with $\Delta z \approx 3$ mm. We provide $T_{1\rho }$ for both tissues, and the average $T_1$ and $T_2$ values (see Table S1).

Figure 5 follows the same structure as Fig. 4, but with a horse tooth. Again, DiSLoP fails to form an image showing the hardest tissues. Only the lower, softer part of the tooth is visible ($T_2 \approx 6$ ms). The PreSLoP reconstruction is significantly better for a comparable scan time. In order to reach the SNR of the PETRA acquisition, PreSLoP would need $\approx 35$ min, i.e. a factor $\times 2$ faster than PETRA.

### Dental phantom

**Figure 6:**
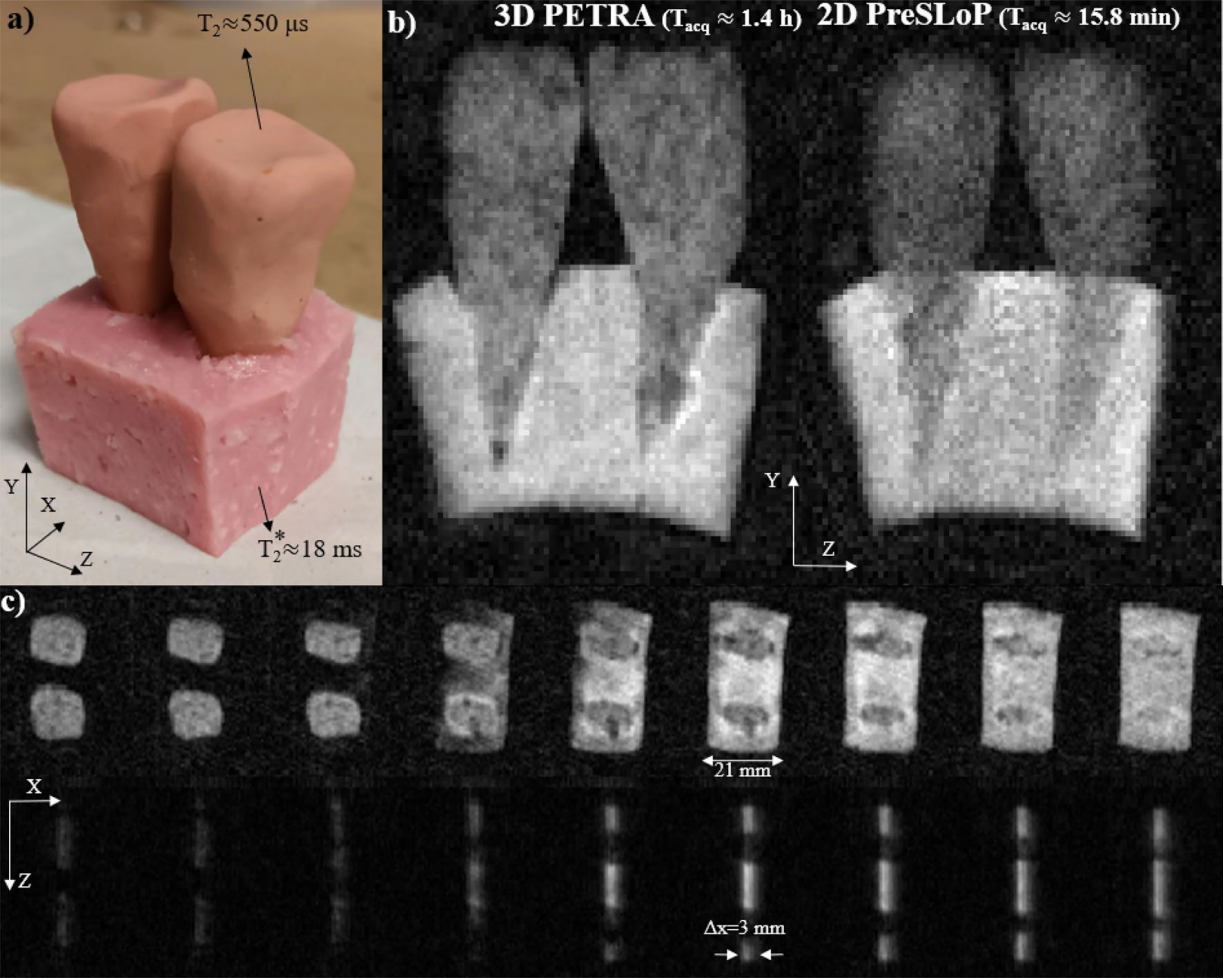
Performance of PreSLoP for a sample containing two clay tooth molds ($T_2\approx 550\,\upmu \hbox {s}$) embedded in a piece of ham ($T_2^*\approx 18 \,\hbox {ms}$). (**a**) Photograph of the sample. (**b**) 3D-PETRA (left) and 2D-PreSLoP (right). (**c**) 2D images for PETRA (top) with slice resolution $\approx 3$ mm, and PreSLoP (bottom) with $\Delta z \approx 3$ mm.

Figure 6 shows 3D images of a sample containing two clay tooth molds ($T_2\approx 550\,\upmu \hbox {s}$) embedded in a piece of ham ($T_2^*\approx 18\,\hbox {ms}$). These results demonstrate the potential applicability of slice selection with PreSLoP for clinical use, e.g for musculoskeletal or dental imaging, where biological samples often include tissues of vastly different properties.

## Discussion

### Slice-selective ZTE imaging

The images in Figs. 2, 3, 4, 5 and 6 demonstrate that spin-locking allows for slice-selective ZTE of ultra-short $T_2$ samples, which was the main goal of this work. The proposed pulse sequences seem to be robust and versatile enough for broad clinical applicability.

We have shown that DiSLoP can image samples where the measured $T_2$ is $\gtrsim 1$ ms, but it is otherwise unsuitable. The main advantage of PreSLoP is its suppressed immunity to $T_{2}^*$ decay after the spin-locking block, allowing for higher SNR than DiSLoP and highlighting the critical importance of the preservation block for imaging hard biological tissues, solid-state matter, and samples with short-lived MR signals.

Sequence performance is strongly dependent on sample properties ($T_{1\rho }$ and $T_2$), the sought image contrast and resolution, and on scanner characteristics (e.g. Eddy currents). In this work, we optimized the pulse sequence parameters through calibration experiments prior to imaging, especially to determine $T_{1\rho }$ and the $B_\text {1SL}$ strength that saturates $T_{1\rho }$ (see Fig. S3). In general, we observe a significant enhancement of $T_{1\rho }/T_2$ for $B_\text {1SL}>{25}\,\upmu \hbox {T}$, and some enhancement already for ${10}\,\upmu \hbox {T}$, especially for ultra-short $T_2$ samples. Regarding the SL pulse duration, we have found that Eq. (2) provides a reasonable estimation for the minimum $t_\text {SL}$ required for slice selection, even if for *ex vivo* hard biological tissues we used shorter times because their short $T_{2\rho }$ values speed up the process. In this sense, we found it useful to search experimentally for the shortest $t_\text {SL}$ required for every sample, which was then confirmed to slice-select adequately with a 3D-PreSLoP acquisition.

DiSLoP is a simpler sequence than PreSLoP, and potentially faster because it can be made immune to the finite dead time ($t_{\text {dead}}$) required to switch the electronics from transmission (Tx) to reception (Rx) mode, thus avoiding the Cartesian single-point k-space gap filling in PETRA. Instead, in DiSLoP one can start the acquisition while $g_\text {RO}$ is ramped (as in UTE), so that acquisitions start at $k=0$ and pointwise encoding is unnecessary. Unfortunately, this possibility requires accurate control and calibration of Eddy currents for different values of $g_\text {RO}$ in each repetition, so for the results above we used the simpler DiSLoP sequence shown in Fig. 1, which includes a pointwise-encoding stage.

One aspect to be aware of is that our proposed sequences are more sensitive to Eddy current effects than PETRA. Consequently, the samples in the PreSLoP and DiSLoP images in Figs. 4, 5 and 6 appear slightly smaller than in the PETRA reconstructions. This is consistent with small Eddy currents generated during the ramps of the SL and RO gradients (Fig. 1), which change the nominal gradient strength and affect the size of the imaged field of view.

### Scan time and SNR

Here we discuss the advantage of PreSLoP with respect to DiSLoP and PETRA in terms of SNR efficiency and scan time. The underlying reasons are rather subtle and parameter-dependent, but some insight can be gained by comparing the three sampling schemes in k-space. Two important concepts in this regard are: (i) scan time in these sequences can be dominated by the number of pointwise acquisitions, especially at low field strengths, where switching from Tx to Rx is slower; and (ii) the largest contribution to the reconstruction SNR comes from the central region of k-space.

**Figure 7:**
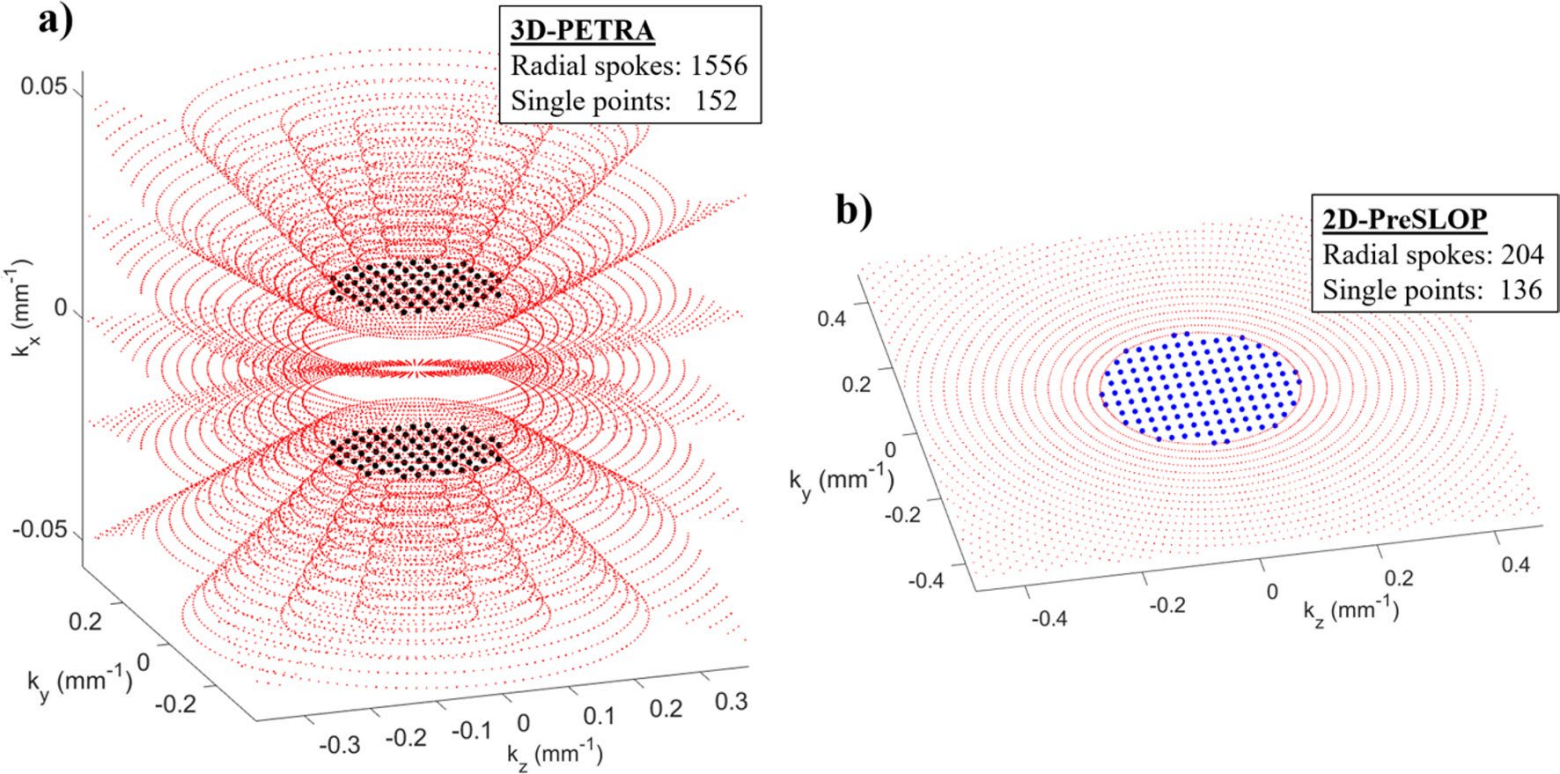
Zoom into the central region of k-space of the cow bone images presented in Fig. 4. (**a**) PETRA. (**b**) PreSLoP.

For illustration purposes, in Fig. 7 we plot k-space positions (not values) for the PETRA and 2D-PreSLoP sequences used to image the bovine femur (Fig. 4), highlighting the pointwise encoding regions (larger black/blue points). A priori, one may expect that the number of pointwise acquisitions in PETRA must be much larger than in 2D-PreSLoP, since the gap in the former is spherical and only a 2D surface in the latter. However, for the chosen slice direction, dead time and resolution, our PETRA implementation (for an even number of slices) requires pointwise sampling only at $k_z=\pm \delta k_z/2$. Consequently, the amount of pointwise acquisitions is similar for PETRA and 2D-PreSLoP (152 vs. 136, see Tab. 1). A slightly different configuration may have required three pointwise planes for PETRA, and 2D-PreSLoP would have scaled even more favorably.

Compared to 2D-PreSLoP, 2D-DiSLoP requires significantly fewer points for a similar scan time (136 vs. 40). This is because the k-space gap in DiSLoP is smaller, since the readout gradient starts to ramp when the magnetization is already transversal, whereas it is already at its full strength during the final excitation pulse in PreSLoP.

In terms of SNR, 2D-DiSLoP always suffers $T_2^*$ decay, and it is to be expected that it performs worse than 2D-PreSLoP for the same scan time. The comparison with PETRA is less straight-forward. For instance, the SNRs of the bovine images are very similar, but PETRA takes $\times 5$ longer than 2D-PreSLoP. As mentioned above, the SNR has a heavy bias towards the number of central points in k-space, and the pointwise acquisitions are comparable (152 for PETRA vs 136 for 2D-PreSLoP). Hence, a PETRA sequence will need to fill a 3D k-space with many radial spokes, while a 2D-PreSLoP sequence will only fill a 2D k-space (1556 vs 204 spokes). This explains why 15 averages of PETRA and 2D-PreSLoP yield similar SNR values, but PETRA takes much longer. Note that the SNR could be overestimated in PreSLoP and DiSLoP reconstructions with rectangular SL pulses, since off-slice contributions act to artificially increase pixel brightness. However, this effect is negligible for long enough $t_\text {SL}$ times (see Eq. 2).

To sum up, it is difficult to predict the efficiency of the different sequences for specific cases, but there are two main trends: i) for samples with longer $T_2$, DiSLoP may outperform PreSLoP because the former requires a smaller pointwise region (which can be even completely avoided if Eddy currents are under control), while PreSLoP is best suited for ultra-short $T_2$ samples; and ii) 2D-PreSLoP should yield a higher SNR than PETRA per unit scan time, because PreSLoP has a 2D scaling for the pointwise region, and because PETRA’s 3D filling of non-central k-space contributes much less to the SNR. Note, however, that PETRA delivers higher SNR if one normalizes by unit volume as well as unit time (see Table 1), so it is probably preferable over multiple 2D-PreSLoP acquisitions for imaging a 3D field of view.

Finally, with regard to flip angles and TR, the TR for both PETRA and slice-selective acquisitions was chosen as the one that optimized the SNR of PETRA images for $90^{\circ }$ excitation, and was mostly dependent on the $T_1$ values of the tissues in the sample. This same TR was then used for the slice-selective sequences, as we found it was also close to optimal. In terms of flip angles, while 2D-DiSLoP inherently uses a $90^{\circ }$ excitation, the SNR of both PETRA and PreSLoP would benefit from choosing the Ernst angle of a given tissue. For tissues with longer $T_2$ this would make for a more equitable comparison between 2D-DiSLoP and 2D-PreSLoP, but since the interest of our work is in ultra-short $T_2$ tissues, our conclusions comparing PETRA and PreSLoP still hold.

### Dynamically decoupled PreSLoP

**Figure 8:**
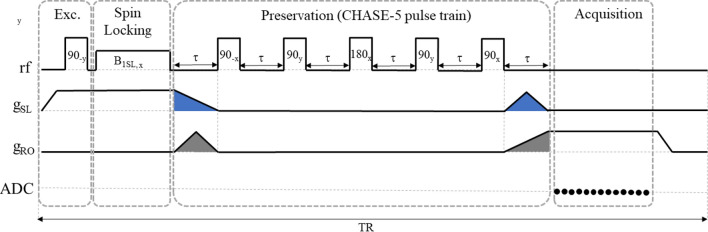
Possible embodiment of PreSLoP where the preservation block includes a CHASE pulse train of length 5, allowing for zero dead time before data acquisition.

We have recently disclosed^32,33^ a possible alternative to the preservation block shown in Fig. 1 of the main text, based on a CHASE-5 sequence^34^. This is shown in Fig. 8 and is basically a modification of the well-known WAHUHA sequence^35^, with a 180$^{\circ }$ rephasing pulse in the middle. This sequence undoes the dephasing effects from both homogeneous (spin-spin interactions) and inhomogeneous (gradient) sources. In our case, it can be used to avoid $T_2^*$ decay during the preservation block, while the dephasing caused by ramping gradients is compensated with the waveforms shown in the figure. Because CHASE-5’s central 180$^{\circ }$ pulse changes the effective sign of spin evolution, the blue area after preservation block in Fig. 8 compensates the ramping down of $g_\text {SL}$. In the same way, the gray area before the preservation block compensates the ramping up of encoding gradients $g_\text {RO}$. Because this sequence compensates gradient evolution, the acquisition starts at $k=0$, eliminating the central gap in *k*-space and therefore the need for pointwise encoding in PreSLoP. This should lead to reduced scan times at the expense of more complicated RF pulse trains and potentially increased SAR (specific absorption rate)^36^.

## Conclusions

We have presented two protocols that combine slice selection through spin-locking with Zero Time Echo 2D imaging. For sub-millisecond $T_2$ tissues, we have presented PreSLoP, which adds a preservation pulse and is capable of 2D imaging the hardest tissues in the body: bone and teeth. PreSLoP suffers from dead time, and thus leaves a central k-space gap that has to be filled by Cartesian pointwise encoding, as in standard PETRA, but has a favorable SNR and 2D scaling, which can lead to significant scan time reduction. DiSLoP is especially suited to moderately short $T_2$ tissues (in the order of few milliseconds), and has the advantage that it can avoid TxRx switching dead time. All in all, we have shown sequences that can combine superior slice selection (which suffers from $T_{1\rho }\gg T_2$ decay) with the most efficient imaging sequence available for ultra-short $T_2$ samples (ZTE) and which have enabled 2D imaging of *ex vivo* bone and teeth.

A potentially interesting alternative to the PreSLoP preservation block (Fig. 1) would include a CHASE-5 dynamical decoupling sequence^34^, which can undo both homogeneous (spin-spin) and inhomogeneous (gradient) dephasing. Because it neutralizes $T_2^*$ decay and compensates gradient evolution, the acquisition could then start at $k=0$, eliminating the central gap in k-space and therefore the need for pointwise encoding. This would lead to a reduced scan time, albeit at the expense of more complicated RF pulse trains and potentially increased specific absorption rates^36^. We note that RF energy deposition can be reduced by a pulsed spin-locking scheme, i.e. a string of short RF pulses instead of continuous irradiation, which can be made equally efficient under some circumstances^37^. This can be of interest both for CHASE-5 based PreSLoP and for simple PreSLoP at higher field scanners.

Finally, the proposed 2D imaging protocols should be compatible also with UTE approaches for moderately short T2 tissues, and they may be advantageous over the double half-pulse RF excitation for slice selection^18^: (i) our protocols are faster by design, since they do not require two acquisitions for every k-space line; and (ii) signal decay during slice selection with PreSLoP and DiSLoP is subject to $T_{1\rho }\gg T_2^*$, and thus easier to deal with than with 2D-UTE in terms of timing and hardware.

## Methods

All experiments in this work have been performed in a 260 mT custom-built DentMRI - Gen I system described elsewhere^8^. Images are reconstructed with Algebraic Reconstruction Techniques (ART)^38,39^, which do not require density compensation and allow for highly undersampled k-space data^8^. $T_1$ characteristic times have been measured by inversion recovery^40^ and $T_2$ times by spin-echo^41,42^, both by fitting single or double exponential functions (see Table S1). Magnetic field inhomogeneities can be shimmed down to the 10 ppm level with small gradient corrections in the 260 mT system. This corresponds to $T'_2\sim 10$ ms, so $T_2^*$ is governed by the shorter $T_2$ (except for ham, where $T_2\approx 75$ and thus $T_2^*\approx 18$ ms).

PreSLoP and DiSLoP are conceived as 2D imaging sequences. Nevertheless, we show also images taken with sequence variants where the slice selection gradient is used for encoding as well. In this way, a 3D image of the slice-selected sample is obtained, which can be useful to benchmark the efficiency of the slice selection block. We call these 3D-PreSLoP and 3D-DiSLoP for clarity. We also find it useful to run the 1D versions of our sequences for fast characterization of slice profiles of simple, homogeneous samples.

Where given, SNR values are estimated by averaging in three different image regions with and without sample (see Supp. Inf. Sect. 2.4).

### Structured phantoms

After investigating the basic control and calibration procedures to slice-select (Supp. Inf. Sects. 2.1 and 2.2), we tested their imaging potential with structured phantoms (see Fig. 2). To demonstrate control over the slice position, a PLA phantom with rectangular holes was filled with 3 % CuSO$_{4}$ doped water, with $T_2\approx 2.5$ ms, long enough for DiSLoP. We compared the $y=0$ slice from a 3D-PETRA acquisition with 3D-DiSLoP reconstructions where slice selection is along the *z* direction, namely at $z=-8$, −4.5, 0, 4.5 and 8 mm (corresponding to detunings of −20.4, −11.5, 0, 11.5, 20.4 kHz with respect to the bare Larmor frequency). Here we use $B_\text {1SL} \approx 90\,\upmu \hbox {T}$, $g_\text {SL}\approx 60$ mT/m, $t_\text {SL} = {800}\,\upmu \hbox {s}$ and $\Delta z \approx 3$ mm.

To show control over the slice thickness we used a similar PLA phantom with a slightly different hole structure, also filled with 3 % CuSO$_{4}$ doped water. The sequence parameters in this case were $g_\text {SL}\approx 60$ mT/m and $t_\text {SL} = 1\,\hbox {ms}$, with $B_\text {1SL}$ values ranging from ${30}\,\upmu \hbox {T}$ (1 mm) to ${120}\,\upmu \hbox {T}$ (4 mm) in steps of ${30}\,\upmu \hbox {T}$.

To characterize the slice-selection dynamics as a function of the sample $T_2$ and SL parameters, and furthermore determine the reliability of Eq. (2) as a predictor of the SL time required to form the slice, we prepared a phantom with a piece of York ham ($T_2\approx 18$ ms), honey ($T_2\approx 1.5$ ms) and clay ($T_2\approx 550\,\upmu \hbox {s}$), and followed the evolution of the different slice profiles for two sets of SL parameters: $g_\text {SL}\approx 35$ mT/m and $B_\text {1SL}\approx 35\,\upmu \hbox {T}$ ($\Delta x\approx 2$ mm); and $g_\text {SL}\approx 60$ mT/m and $B_\text {1SL}\approx 90\,\upmu \hbox {T}$ ($\Delta x\approx 3$ mm).

### Biological samples

**Table 1:** Image acquisition parameters. “NA” and “NR” stand for “not applicable” and “not relevant” respectively.

Image	Sequence	spin-locking parameters {$B_\text {1SL}$, $g_\text {SL}$, $t_\text {SL}$} {$\mu$T, mT/m, $\mu$s}	Nyquist undersampling	FOV (mm$^3$)	Pixel size (mm$^3$)	Dead time (us) / Acquisition time (us)	Bandwidth (kHz)	TR (ms)	Radial spokes	Single points	Averages	Scan time (min)	SNR
Fig.4(b)Left	PETRA	NA	8	46$\times$34$\times$30	1$\times$1$\times$1	80 / 600	38.3	75	572	96	25	20.88	NR
Fig.4(b)Right	3D-PreSLoP	{150, 100, 100 }	8	46$\times$34$\times$30	1$\times$1$\times$1	80 / 600	38.3	75	572	96	25	20.88	NR
Fig.4(c)Left	PETRA	NA	2	46$\times$34$\times$30	0.5$\times$0.5$\times$3	90 / 600	76.6	75	1556	152	15	32.03	3.8
Fig.4(c)Middle	2D-PreSLoP	{150, 100, 100 }	2	46$\times$34	0.5$\times$0.5$\times$3	90 / 600	76.6	75	204	136	15	6.34	3.6
Fig.4(c)Right	2D-DiSLoP	{150, 100, 100 }	2	46$\times$34	0.5$\times$0.5$\times$3	NA / 600	76.6	75	204	44	15	4.65	1.0
Fig.5(b)Left	PETRA	NA	8	38$\times$90$\times$21	1$\times$1$\times$1	110 / 600	75	25	764	288	375	164.37	NR
Fig.5(b)Right	3D-PreSLoP	{90, 60, 190 }	8	38$\times$90$\times$21	1$\times$1$\times$1	110 / 600	75	25	764	288	375	164.37	NR
Fig.5(c)Left	PETRA	NA	2	38$\times$90$\times$21	1$\times$1$\times$3	110 / 600	75	25	1010	112	150	70.12	6.2
Fig.5(c)Middle	2D-PreSLoP	{90, 60, 190 }	2	38$\times$90	1$\times$1$\times$3	110 / 600	75	25	200	112	150	19.37	4.7
Fig.5(c)Right	2D-DiSLoP	{90, 60, 190 }	2	38$\times$90	1$\times$1$\times$3	NA / 600	75	25	200	24	150	14.00	1.2
Fig.6(b)Left	PETRA	NA	2	42$\times$50$\times$39	0.5$\times$0.5$\times$3	110 / 550	91	100	2122	420	20	84.7	NR
Fig.6(b)Right	2D-PreSLoP	{180, 120, 550 }	2	42$\times$50	0.5$\times$0.5$\times$3	100 / 550	91	100	222	252	20	15.8	NR
Fig.6(c)Up	PETRA	NA	12	45$\times$52$\times$39	0.68$\times$2$\times$0.65	100 / 600	55	75	1082	336	20	35.45	NR
Fig.6(c)Down	3D-PreSLoP	{90, 60, 500 }	12	45$\times$52$\times$39	0.68$\times$2$\times$0.65	NA / 600	25	75	1082	48	38	53.7	NR

To test the performance and potential applicability of PreSLoP and DiSLoP sequences, we also ran acquisitions on hard biological samples and compared them to PETRA in terms of SNR and scan time for the same slice and in-plane resolution. For a first ex vivo test we used a piece of cortical bone from a bovine femur ($T_2 \approx 440\,\upmu \hbox {s}$), obtained from a local butcher and stripped off of the surrounding soft tissues. The second experiment employed a horse tooth stripped off of soft tissues and dehydrated to remove water residues in internal cavities. The average $T_2$ for this sample was only around ${260}\,\upmu \hbox {s}$. These images were taken with $t_\text {SL} \ll 7\pi /2\Omega _\text {1SL}$. All sequence parameters are given in Table 1 for quick reference. For the 2D-DiSLoP acquisitions, the gradient ramps lasted $100\,\,\upmu \hbox {s}$, so data acquisition started ${400}\,\,\upmu \hbox {s}$ after spin-locking.

### Dental phantom

Finally, we studied the performance of PreSLoP for slice selection of an ultra-short $T_2$ sample in the presence of softer matter, which is more representative of clinical conditions. To this end, we prepared a mock-up of a dental phantom with two clay “teeth” ($T_2\approx 550\,\,\upmu \hbox {s}$) embedded onto a piece of ham ($T_2^*\approx 18\,\hbox {ms}$) that emulated the gums. Details on the sequence parameters are provided in Table 1.

### Ethical approval and informed consent

All animal parts were obtained by means Clinical Veterinary Hospital of Cardenal Herrera University and research was conducted 3R principles.

### Supplementary Information

Supplementary Information 1.

## Data Availability

All datasets and reconstruction and postprocessing methods generated or used during the present study are available from the corresponding author on reasonable request.
