# Author Correction: Low field slice-selective ZTE imaging of ultra-short *T*_2_ tissues based on spin-locking

**DOI:** 10.1038/s41598-023-46700-0

**Published:** 2023-11-15

**Authors:** Jose Borreguero, Fernando Galve, José M. Algarín, José M. Benlloch, Joseba Alonso

**Affiliations:** 1 Tesoro Imaging S.L., 46022 Valencia, Spain; 2 Institute for Molecular Imaging and Instrumentation, Spanish National Research Council, 46022 Valencia, Spain; 3 Institute for Molecular Imaging and Instrumentation, Universitat Politècnica de València, 46022 Valencia, Spain

Correction to: *Scientific Reports* 10.1038/s41598-023-28640-x, Published online 30 January 2023

The Funding section in the original version of this Article was incomplete.

“This work was supported by the Ministerio de Ciencia e Innovación of Spain through research grant PID2019-111436RB-C21.”

now reads:

“This work was supported by the Ministerio de Ciencia e Innovación of Spain through research grant PID2019-111436RB-C21 and by the Generalitat Valenciana through grant CIPROM/2021/003.”

The original Article has been corrected.

