# Supplementary material for: Low field slice-selective ZTE imaging of ultra-short T2 tissues based on spin-locking
Source: Sci Rep. 2023 Jan 30;13:1662. doi: 10.1038/s41598-023-28640-x (PMC9886919; doi:10.1038/s41598-023-28640-x)
Supplement: Supplementary file 1 — Supplementary Information 1. [file 41598_2023_28640_MOESM1_ESM.pdf]

Supporting Information for: Low field slice-selective ZTE imaging of ultra-short T_2 tissues based on spin-locking

Jose Borreguero[1*], Fernando Galve[2,3], José M. Algarín[2,3], José M. Benlloch[2,3] and Joseba Alonso[2,3*]

[1]Tesoro Imaging S.L., Valencia, 46022, Spain.
[2]Institute for Molecular Imaging and Instrumentation, Spanish National Research Council, Valencia, 46022, Spain.
[3]Institute for Molecular Imaging and Instrumentation, Universitat Politècnica de València, Valencia, 46022, Spain.

*Corresponding author(s). E-mail(s): pepe.morata@tesoroimaging.com; joseba.alonso@i3m.upv.es;

1 Spin-locking theory

1.1 Spin-locking equations

The equations that govern the magnetization at each infinitesimal plane in the slice selection direction (z) are:

$$\begin{aligned} M_x(t) &= M_0 \cos^2\alpha\, \mathrm{e}^{-t/T_{1\rho}} + M_{\mathrm{eq.}} \cos\alpha (1 - \mathrm{e}^{-t/T_{1\rho}}) + M_0 \sin^2\alpha \cos(\gamma B t)\, \mathrm{e}^{-t/T_{2\rho}} \\ M_y(t) &= -M_0 \sin\alpha \sin(\gamma B t)\, \mathrm{e}^{-t/T_{2\rho}} \\ M_z(t) &= M_0 \sin\alpha \cos\alpha (\mathrm{e}^{-t/T_{1\rho}} - \cos(\gamma B t) \mathrm{e}^{-t/T_{2\rho}}) + M_{\mathrm{eq.}} \sin\alpha (1 - \mathrm{e}^{-t/T_{1\rho}}), \end{aligned} \tag{1}$$

which for $t \ll T_{1\rho}$ become

$$\begin{aligned} M_x(t) &= M_0 \cos^2\alpha + M_0 \sin^2\alpha \cos(\gamma B t)\, \mathrm{e}^{-t/T_{2\rho}}, \\ M_y(t) &= -M_0 \sin\alpha \sin(\gamma B t)\, \mathrm{e}^{-t/T_{2\rho}}, \\ M_z(t) &= M_0 \sin\alpha \cos\alpha (1 - \cos(\gamma B t) \mathrm{e}^{-t/T_{2\rho}}), \end{aligned} \tag{2}$$

with $B = \sqrt{B_{1\mathrm{SL}}^2 + (g_{\mathrm{SL}} \cdot z)^2}$, $\cos\alpha = B_{1\mathrm{SL}}/B$, $\sin\alpha = g_{\mathrm{SL}} \cdot z/B$, with $B = B(z)$ and $M_{\mathrm{eq.}}$ is the equilibrium magnetization corresponding to $B(z)$. They can be obtained by a simple transformation to the rotating frame, and then calculating usual evolution around a fixed B (at each z-plane) with $T_{1\rho}$ and $T_{2\rho}$ replacing T_1 and T_2. These expressions are valid in the frame rotating at γB_0, assuming the excited slice is at $z = 0$. The transverse decay rate, $T_{2\rho}$, is given by [1,2]

$$\frac{1}{T_{2\rho}} \simeq \frac{1}{2}\left(\frac{1}{T_1} + \frac{1}{T_2}\right), \tag{3}$$

which for $T_1 \gg T_2$ (where SL is potentially valuable) leads to $T_{2\rho} \simeq 2T_2$. The main idea behind slice selection through SL is that for short $T_{2\rho}$ and/or a strong slice selection gradient (short T_2^*), only the average magnetization in the selected (spin-locked) slice remains.

For tissues with fast $T_{2\rho}$, oscillatory terms vanish quickly and $\vec{M} \approx (M_0 \cos^2\alpha, 0, M_0 \sin\alpha\cos\alpha)$, leading to an observable (i.e. transverse) magnetization

$$M_{\mathrm{obs}} = |M_x + \mathrm{i}M_y| \approx M_0 \cos^2\alpha = M_0 \frac{B_{1\mathrm{SL}}^2}{B_{1\mathrm{SL}}^2 + (g_{\mathrm{SL}} \cdot z)^2}, \tag{4}$$

which is Lorentzian with a full width at half maximum of $\Delta z \approx 2B_{1\mathrm{SL}}/g_{\mathrm{SL}}$ (Eq. (1) in main text). The ensemble average in a thin slice at $z = 0$ has almost negligible average M_z if the sample's slice is homogeneous enough, since $\sin\alpha$ is an odd function.

For tissues with long $T_{2\rho}$, one can imagine the sample as divided in slices of width Δz, whose average magnetization is detected. For slices far from the selected one, $B(z) \approx |g_{\mathrm{SL}} \cdot z|$ and the oscillatory term in $M_x(t)$ has a slice-average

$$\int_{z_0-\Delta z/2}^{z_0+\Delta z/2} \cos\gamma Bt \approx \mathrm{sinc}(\gamma g_{\mathrm{SL}} \Delta z\, t_{\mathrm{SL}}/2)\cos(\gamma g z_0 t),$$

where we have neglected the z-dependence of $\sin^2\alpha$, and z_0 is the center for this slice. Thus the envelop decays as $\mathrm{sinc}(\gamma g_{\mathrm{SL}}\Delta z\, t_{\mathrm{SL}}/2) = \mathrm{sinc}(\gamma B_{1\mathrm{SL}} t_{\mathrm{SL}})$. For instance, for $t_{\mathrm{SL}} \gtrsim 7\pi/(2\gamma B_{1\mathrm{SL}})$, a fraction $\lesssim 10\,\%$ survives (Eq. (2) in main text). The same argument applies for the oscillatory term in $M_z(t)$.

Of course, samples are never homogeneous, and the dynamics near the selected slice are more complicated for long $T_{2\rho}$, but the derived expressions give an approximate idea of expected timescales and behavior in the sequences in Fig. 1 of the main text.

1.2 Advanced spin-locking schemes

Sharp slice profiles can be achieved with sinc-modulated SL pulses. *Yan et al.*[3] give a rigorous approach with a multi-order expansion of the magnetization components. They show that (page 10 in their work) when $B_{1\mathrm{SL}}$ is a

sinc function, the non-linear response of spins is separated into 'resonant' and off-resonant regions, given by the spectral width of the sinc function. Their solution is

$$M_x + iM_y \sim (1 - \cos\gamma B_1(\omega))/2 + e^{-i\omega t}(1 + \cos\gamma B_1(\omega))/2,$$

with $\omega = \gamma g z$ and $B_1(\omega)$ the Fourier transform of the RF pulse:

$$\gamma B_1(\omega) = \gamma\mathcal{F}[B_1 \mathrm{sinc}(\frac{\gamma g_{\mathrm{SL}}\Delta z t}{2})] = \pi\frac{2B_1}{g_{\mathrm{SL}}\Delta z}\mathrm{rect}(\frac{\omega}{\gamma g_{\mathrm{SL}}\Delta z}) = \pi\frac{B_1}{B_{1\mathrm{SL}}}\mathrm{rect}(\frac{\omega}{\gamma g\Delta z}),$$

where in the last step we have used the definition of $B_{1\mathrm{SL}}$ in eq.(1) of main text. Thus, within their approximation, off-resonance spins simply dephase through an evolution of the form $M_x + iM_y \sim e^{-i\gamma g z t}$ for each z. On-resonance spins follow a mild dephasing at the end of spin-locking unless we used $B_1 = B_{1\mathrm{SL}}$, in which case $M_x + iM_y \sim 1$ inside the region $\|\omega\| \leq \gamma g_{\mathrm{SL}}\Delta z/2$.

Thus three consequences follow: (i) off-slice spins dephase much faster than in the constant $B_{1\mathrm{SL}}$ case; (ii) off-slice and in-slice regions are defined by the argument in the sinc function; and (iii) depending on the strength of $B_1/B_{1\mathrm{SL}}$, in-slice spins feel an oscillatory behaviour and partial dephasing that has to be taken into account. In Fig. 2 we see that off-slice spins fall much more abruptly than in the constant $B_{1\mathrm{SL}}$ case, proving (i). Also, in our experiments and simulations (not shown) we have checked that for a sinc with a fixed spectral width but increasing B_1, the approximately rectangular profile of the selected slice is kept (ii), but the amplitude of the selected slice changes with B_1, leading also to oscillatory in-slice profiles. A more detailed analysis and comparison with theory is beyond the scope of this paper, and is left for future work.

Analogously, multi-slice selection occurs when the time-profile of $B_{1\mathrm{SL}}(t)$ has a Fourier transform with two peaks, which is achieved by a double-tone $B_{1\mathrm{SL}}(t) = B_1\cos(\gamma(B_0 + g_{\mathrm{SL}}\, z_1)t) + B_2\cos(\gamma(B_0 + g_{\mathrm{SL}}\, z_2)t)$. Here $B_1(\omega)$ is the sum of two sinc function centered at those frequencies, and thus, according to the arguments above, the regions where most dephasing occurs are those where $B_1(\omega) \sim 0$, far from the two peaks. Another way to reason this situation is by moving to a frame rotating at $\gamma(B_0 + g_{\mathrm{SL}}\, z_1)$, with a field:

$$\begin{aligned}\vec{B} = {} & g_{\mathrm{SL}}(z - z_1)\hat{u}_z + \frac{B_1}{2}\hat{u}_x + \\ & \frac{B_2}{2}(\hat{u}_x\cos[(\omega_1 - \omega_2)t] + \hat{u}_y\sin[(\omega_1 - \omega_2)t]).\end{aligned} \tag{5}$$

For spins near z_1 and when $\omega_1 - \omega_2 = \gamma g_{\mathrm{SL}}(z_1 - z_2) \gg \gamma B_1$, the oscillatory terms are averaged out in a timescale $\propto 2\pi/(\gamma B_1)$ (relevant to spin-locking), resulting in $\vec{B} = g_{\mathrm{SL}}(z - z_1)\hat{u}_z + \frac{B_1}{2}\hat{u}_x$. This locks spins near z_1 and dephases the rest. Repeating the argument in the frame rotating at $\gamma(B_0 + g_{\mathrm{SL}}\, z_2)$, we obtain $\vec{B} = g_{\mathrm{SL}}(z - z_2)\hat{u}_z + \frac{B_1}{2}\hat{u}_x$ which spin-locks near z_2. As an example, consider a

Table 1 Relaxation parameters of employed samples and figures where they have been used.

Parameter	T_1 (ms)	T_2 (ms)	Figures
York ham	289 ms	75 ms ($T_2^* \approx$ 18 ms)	3, 6, S1
1% $CuSO_4$ water	16 ms	7.5 ms	S1
3% $CuSO_4$ water	3 ms	2.5 ms	2, S1, S2, S3, S4
Honey	14.4 ms	1.5 ms	3, S1
Eraser	27 ms	800 µs	S1
Photopolymer resin	23.1 ms	650 µs	S1
Clay	36 ms	550 µs	3, 6, S1, S5
Bone	730 ms	315 / 630 µs	4, S1, S6
Teeth	329 ms	275 µs / 6 ms	5, S1, S6

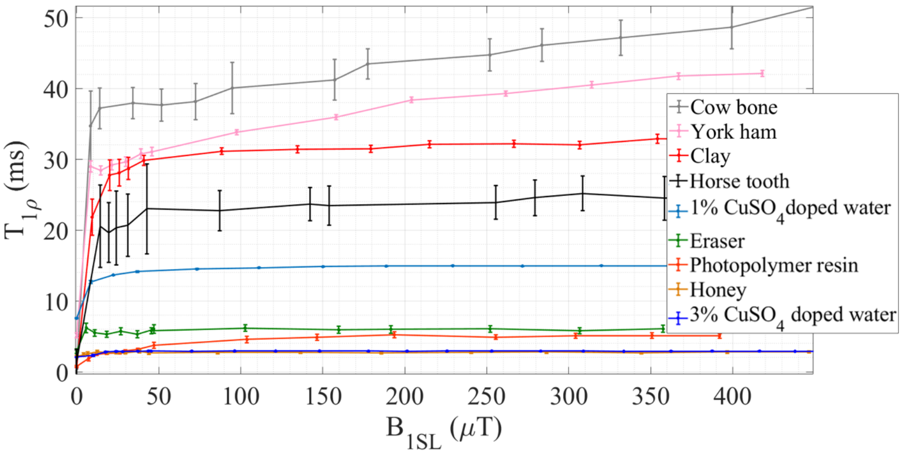

Fig. 1 $T_{1\rho}$ as a function of the applied locking RF field B_{1SL} for some of the samples employed throughout this work. $T_{1\rho}$ values associated to $B_{1SL} = 0$ correspond to T_2^*, virtually the same as the T_2 values reported in Tab. S1 except for ham. The lines simply connect the data points and are there only for readability.

slice selection gradient of 70 mT/m and a spin-locking amplitude that selects a slice with $\Delta z = 1$ mm (so that $B_1 = g_{SL}\,\Delta z/2$). The characteristic evolution is given mainly by $\gamma B_1 \approx 2\pi \cdot 1.5$ kHz, while oscillatory terms evolve at $\omega_2 - \omega_1 \approx 2\pi \cdot 30$ kHz for double-tone spin-locking with $|z_1 - z_0| = 1$ cm, and thus average to zero.

2 Calibration and control experiments

2.1 Measured relaxation parameters

Table S1 contains the relaxation parameters measured for the samples used in the main text. T_1 is measured by inversion recovery and T_2 by spin-echo, both by fitting single or double exponential functions where necessary.

We have also measured the dependence of $T_{1\rho}$ on the SL amplitude (B_{1SL}) for the samples used throughout this work (Fig. 1). The main value of these

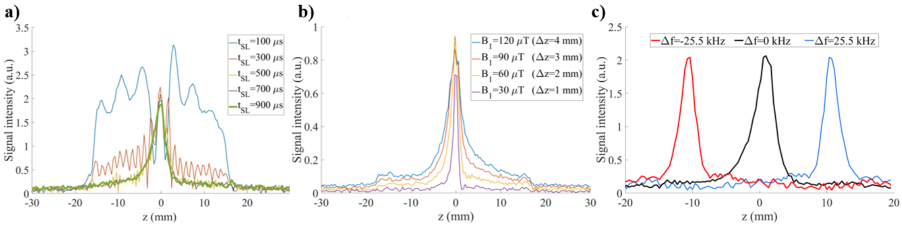

Fig. 2 Control over the spin-locking block with 1D-DiSLoP and a PLA (polylactic acid) cuboid container filled with 3 % $CuSO_4$ doped water. a) Slice profile as a function of t_{SL} ($B_{1SL} \approx 90\,\mu T$, $g_{SL} \approx 60\,mT/m$). b) Slice thickness as a function of B_{1SL} ($t_{SL} = 900\,\mu s$, $g_{SL} \approx 60\,mT/m$). c) Slice position as a function of Larmor detuning ($B_{1SL} \approx 90\,\mu T$, $g_{SL} \approx 60\,mT/m$, $t_{SL} = 900\,\mu s$).

measurements is that it is hard to predict $T_{1\rho}$ based on any hard/soft material distinction, and the B_{1SL} strength that saturates $T_{1\rho}$. Thus, we found it useful to carry out these measurements before imaging a new sample to optimize the DiSLoP and PreSLoP sequence parameters. Every data point in Fig. 1 is determined from sequences consisting only of the excitation and spin-locking blocks, where we sweep the duration t_{SL} of the latter. From every value of t_{SL}, we use a single data point (around 1 ms after the beginning of the acquisition, to avoid ring-down), and we fit an exponential model to determine $T_{1\rho}$ from the data set for a given B_{1SL}. This procedure is repeated for all the B_{1SL} values in the plot, where the lines are included merely to guide the eye. As expected, for low B_{1SL} the decay in the SL rotating frame $T_{1\rho}$ tends to T_2^*. For higher B_{1SL} it can increase to a significant fraction of T_1, which is one of the key advantages of the proposed slice selection method. We observe $T_{1\rho} < T_1$ in all samples except for bone, consistent with expectations ($T_{1\rho}$ cannot get much longer than T_1 for soft tissues[1]). Also, note that there are two T_1 and T_2 values for bone and dental samples in Tab. S1, because they consist of two types of tissues, but we only show the average $T_{1\rho}(B_{1SL})$ curve in Fig. 1 to lighten the plot. Nevertheless, for the bone and dental images in the main text, we give both $T_{1\rho}$ values for each sample at the corresponding B_{1SL}.

2.2 Control over slice-selection and preservation blocks

In a first set of experiments we demonstrated experimental control over the slice selection process with 1D-DiSLoP and a cuboid container filled with 3 % $CuSO_4$ doped water (T_1 and T_2 in Tab. S1). Here, the hard 90° RF pulses were 10 μs long with an amplitude $\approx$ 550 μT, $B_{1SL} \approx 90\,\mu T$ and $g_{SL} \approx 60\,mT/m$. We studied initially the *spin-locking time* required for slice selection. To this end, we used a rephasing block after the slice selection block, and then applied an encoding gradient in the direction of slice selection. To demonstrate control over the *slice thickness* with 1D-DiSLoP, we fixed $t_{SL} = 900\,\mu s$ and $g_{SL} \approx 60\,mT/m$ and used multiple spin-locking field amplitudes ($B_{1SL} \approx$ 30, 60, 90, 120 μT, corresponding to slice thicknesses $\Delta z \approx$ 1, 2, 3, 4 mm). Finally, to show control over the *slice position*, we set

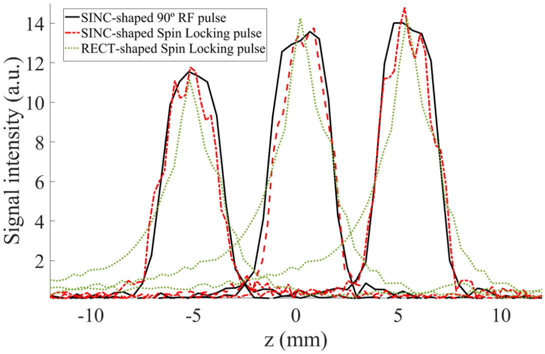

Fig. 3 Comparison between square (green) and sinc-modulated spin-locking (red) and sinc-modulated standard slice selection (black) for the same 3 % $CuSO_4$ doped water sample as in Fig. 2. All curves are normalized to the same height to ease comparison.

$B_{1SL} \approx 90\,\mu T$, $g_{SL} \approx 60\,mT/m$ and $t_{SL} = 900\,\mu s$ ($\Delta z \approx 3\,mm$), and we detuned the SL frequency by $\Delta f = -25.5$, 0 and 25.5 kHz from the Larmor frequency at the gradient isocenter, shifting the selected slice to $z_0 \approx -10$, 0 and 10 mm respectively.

Figure 2 shows the results obtained following the above procedures. Figure 2a shows the 1D profiles for varying t_{SL} and demonstrates control over the slice-selection process. A 3 mm slice is selected after $t_{SL} \gtrsim 500\,\mu s$, not far from the $\approx 460\,\mu s$ predicted by Eq. (2) in the main text. Figure Sreffig:SLcontrolb shows slice-selected Lorentzian profiles for varying B_{1SL} and demonstrates control over the slice thickness. As expected, the off-slice contribution is more prominent for higher Δz. The profile asymmetries observed are compatible with a decaying drift of B_0 caused by Eddy currents. Figure 2c shows slice-selected profiles with three different Δf and demonstrates control over the slice position.

We also investigated the effect of square and sinc-modulated SL pulses on slice shape. The moderate T_2 (≈ 2.5 ms, see Tab. 1) allowed for standard slice selection also (i.e. without spin-locking), with a sinc-modulated excitation and gradient echo encoding with the shortest echo time possible in our setup (2 ms). Both sinc functions included five lobes in total. The amplitude and duration of the rectangular pulses were $B_{1SL} \approx 90\,\mu T$ and $t_{SL} = 900\,\mu s$, $\approx 30\,\mu T$ and 1.4 ms for the sinc pulses. In both cases, $g_{SL} \approx 60\,mT/m$ and $\Delta z \approx 3\,m$. Figure 3 shows 1D profiles of slices selected with square SL, sinc SL and standard methods. Despite be far of linear excitation regime only valid to low flip angle cases, we observe similar profile transitions for both sinc-modulated experiments, and a peaked profile for square SL, as expected. We have normalized the profiles in Fig. 3 to the same peak values for visual aid. However, the standard gradient echo acquisition has a lower signal than the constant SL curve by a factor of 1.5, since the magnetization is lost at a rate given by T_2^* instead of $T_{1\rho}$. The signal is even lower with sinc SL (factor of 3.2), due to the longer SL pulse duration and the shorter effective $T_{1\rho}$.

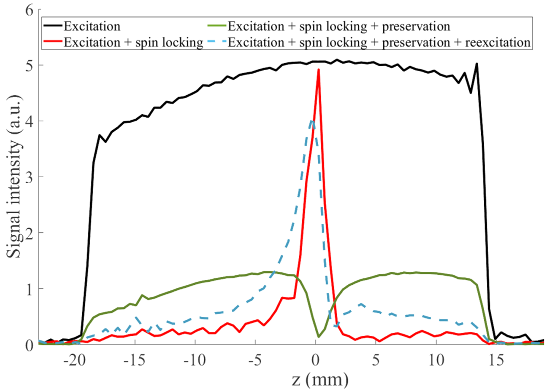

Fig. 4 Profile of the transverse magnetization after each stage in PreSLoP for the same 3 % $CuSO_4$ doped water sample as in Fig. 2, with $\Delta z \approx 2$ mm: after initial 90°_x excitation (black), after spin-locking (red), after 90°_{-x} storage pulse (green), and after final 90°_x excitation (dashed blue). In the latter, there is a delay $\tau = 200\,\mu$s where gradients are ramped between the 90°_{-x} and 90°_x pulses. We have used $B_{1\mathrm{SL}} \approx 60\,\mu$T, $g_{\mathrm{SL}} \approx 60$ mT/m, $t_{\mathrm{SL}} = 1$ ms. The full (black) curve is not flatter due to capillarity effects and bubble formation in the small PLA container.

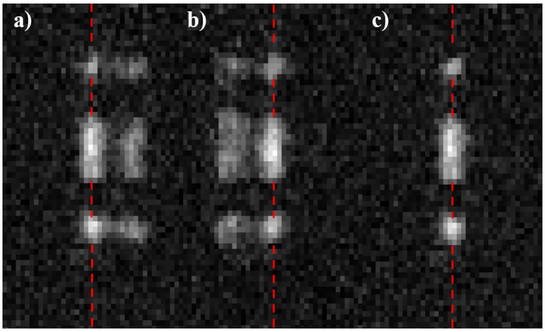

Fig. 5 Effect of the g_{SL} polarity over slice profile definition obtained with 3D-PreSLoP and a clay phantom: a) positive g_{SL} in all repetitions; b) negative g_{SL} in all repetitions; c) alternating g_{SL} polarity between repetitions. Red dotted lines mark the $x = 0$ position in each image, where the slice is nominally selected.

After characterizing the basic experimental control with DiSLoP, we moved on to PreSLoP. To this end, we used again the above sample (3 % $CuSO_4$ solution) and measured the 1D profiles at different steps along the PreSLoP sequence: after the initial 90° excitation, after spin-locking, after the preservation pulse (-90°) and after the final excitation pulse (prior to image encoding). Here we used $B_{1\mathrm{SL}} \approx 60\,\mu$T, $g_{\mathrm{SL}} \approx 60$ mT/m, $t_{\mathrm{SL}} = 1$ ms and $\Delta z \approx 2$ mm. Figure 4 shows the evolution of the magnetization throughout a 1D-PreSLoP sequence. After the preservation pulse, only off-slice components are visible, since in-slice magnetization is stored along z and the transverse contribution is negligible. The profiles after preservation and in the final stage are asymmetric, presumably due to drifting Eddy currents, as in Fig. 2b.

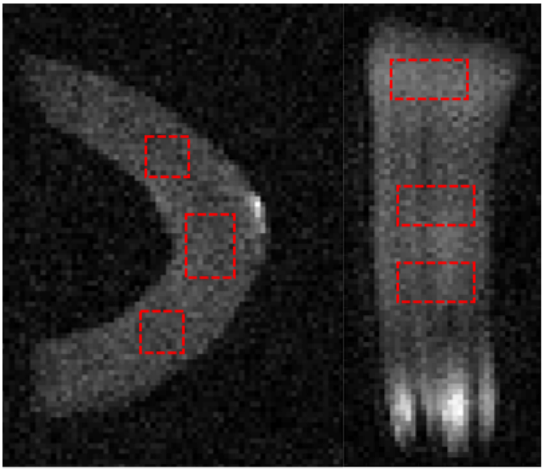

Fig. 6 Regions of interest selected for SNR estimations in Figs. 4-5 of the main text.

2.3 Effect of gradient polarity on PreSLoP performance

PreSLoP is subject to distortions in the slice profile for short repetition times, presumably due to Eddy currents inducing drifts on B_0. Inverting the polarity of the spin-locking gradient (g_{SL}) for each radial acquisition mitigates this effect, because the contributions average out to a large extent. For illustration purposes, Fig. S5 shows the effect of: i) employing a single polarity on a 3D-PreSLoP (positive on the left image, negative on the middle image), where artifacts are clearly visible; and ii) alternating polarity between repetitions, where they are largely suppressed. These images have been taken with a clay phantom.

2.4 SNR estimations

The SNR values given in Figs. 4-5 of the main text are estimated by averaging the signal strength in three bright regions (inside each sample), divided by the average signal strength in a dark region (background). Figure 6 shows the regions of interest selected for the bone sample (left) and the horse tooth (right).

References

1. S. Kelly, C. Sholl, A relationship between nuclear spin relaxation in the laboratory and rotating frames for dipolar and quadrupolar relaxation. Journal of Physics: Condensed Matter **4**, 3317 (1992)

2. A. Wheaton, A. Borthakur, M. Corbo, G. Moonis, E. Melhem, R. Reddy, $T_{2\rho}$-weighted contrast in MR images of the human brain. Magnetic Resonance in Medicine **52**, 1223 (2004)

3. H. Yan, B. Chen, J.C. Gore, Approximate solutions of the bloch equations for selective excitation. Journal of Magnetic Resonance **75**, 83–95 (1987)
